# Defining the critical axial length threshold for transition from physiological growth to pathological fundus structural remodeling in adolescent myopia

**DOI:** 10.3389/fcell.2026.1938535

**Published:** 2026-09-04

**Authors:** Ting Zhou, Xu Zhang, Yajie Zheng, Jie Wang, Yu Jiang, Hanyang Yu, Shitong Huang, Ningfeng Li, Xinrui Zou, Shukui Li, Weihao Bi, Shuoxian Chen, Siyu Liu, Zhimeng Zhang, Feng Shi, Wenzhi Huang, Danna Chen, Hanyue Yang, Xi Wang, Zongyin Gao, Xingye Wang, Yunxia Leng

**Affiliations:** 1 Department of Ophthalmology, The Second Affiliated Hospital, South China University of Technology, Guangzhou First People’s Hospital, Guangzhou, China; 2 EVision Technology (Beijing) Co., Ltd., Beijing, China; 3 Qiqihar Medical University, Qiqihar, Heilongjiang, China

**Keywords:** axial elongation, childhood myopia, Choroid–sclera biomechanics, fundus tessellation density, pathological transition threshold, peripapillary atrophy, posterior pole remodelling, quantitative fundus imaging

## Abstract

The dynamic evolution of fundus tessellation density (FTD) and peripapillary atrophy (PPA) during childhood myopia progression—and their threshold-associated transitions relative to axial length (AL)—remain incompletely characterized. To address this gap, we prospectively quantified longitudinal FTD and PPA changes in a school-aged cohort with progressive myopia, and modeled their nonlinear dose–response relationships with AL to identify the biomechanical threshold at which fundus remodeling shifts from physiological adaptation to early pathological tissue deformation. A cohort of 371 children aged 8–11 years was prospectively followed for 2 years. FTD and PPA were measured via a validated deep learning system; myopia progression subtypes were stratified via k-means clustering by annual AL elongation rate; longitudinal trajectories and inflection points were estimated via linear mixed-effects models and quadratic regression. Three progression phenotypes emerged: stable (0.147 mm/y), moderate (0.443 mm/y), and rapid (0.827 mm/y). Each 1 mm AL increase correlated with 0.127 mm^2^ (95% CI 0.106–0.148) PPA expansion (*p* < 0.001) and 0.011 FTD elevation (*p* < 0.001). PPA showed J-shaped acceleration with inflection at 23.99 mm; FTD showed U-shaped trajectory, declining before 23.66 mm then rising exponentially. This is the first longitudinal study to identify a ∼24 mm (23.66–23.99 mm) biomechanical threshold for early fundus pathology in children—substantially below the adult high-myopia criterion (>26 mm)—supporting a paradigm shift toward earlier AL-driven risk stratification based on tissue biomechanical susceptibility rather than conventional static refractive cutoffs.

## 1 Introduction

Myopia has become a major global public health concern, with particularly high and rapidly increasing prevalence among adolescents in East Asia. Epidemiological models project that by 2050, approximately 4.8 billion individuals—nearly half of the world’s population—will be affected by myopia (Holden et al., 2016). The principal clinical threat posed by this epidemic lies not in refractive error *per se*, but in progressive, irreversible structural remodeling of the posterior pole driven by pathological axial elongation. Such remodeling underlies an elevated lifetime risk of vision-threatening complications, including myopic maculopathy, lamellar or retinal schisis, choroidal neovascularization, and posterior staphyloma (Vagge et al., 2026; Liu et al., 2026).

Traditional clinical assessment of myopia has predominantly relied on spherical equivalent (SE). However, as a composite refractive metric, SE is confounded by multiple anatomical and functional determinants—including corneal curvature, crystalline lens power, and accommodative status—thereby exhibiting intrinsic limitations in sensitivity and temporal resolution for detecting early structural pathology. Consequently, SE provides an indirect and delayed reflection of posterior pole remodeling, rather than a direct, objective measure of ocular structural integrity (Yamashita et al., 2024; Jiang et al., 2024). In contrast, axial length (AL) measurement is widely regarded as the gold-standard biometric parameter for monitoring myopia progression, as it directly quantifies vitreous chamber elongation—the principal anatomical substrate underlying myopic pathological remodeling (Lim et al., 2025; Verhoeven et al., 2015). Nevertheless, AL remains a unidimensional surrogate that cannot capture the spatial heterogeneity or microarchitectural complexity of three-dimensional fundus changes. Critically, no consensus-based, clinically validated AL threshold currently exists to reliably differentiate physiological axial growth from the onset of pathological remodeling in pediatric and adolescent populations.

To advance myopia prevention and control toward earlier, pre-symptomatic stages, there is an urgent clinical need for sensitive, quantitative fundus biomarkers capable of objectively capturing posterior pole structural remodeling—as well as for rigorously defined transition thresholds that delineate the shift from physiological adaptation to incipient pathological change. Fundus tessellation density (FTD) and peripapillary atrophy (PPA) represent the two most prevalent early fundus manifestations in myopic eyes (Lyu et al., 2021; Chen et al., 2023). Fundus tessellation reflects thinning of the retinal pigment epithelium (RPE) and choriocapillaris, serving as an *in vivo* imaging correlate of choroidal microcirculatory insufficiency and biomechanically driven scleral expansion (Gong et al., 2024; Guo et al., 2018). PPA arises from mechanical stretching of the peripapillary sclera during axial elongation; progressive enlargement of the PPA area signifies increasing separation between the RPE–Bruch’s membrane complex and the underlying scleral margin (Guo et al., 2018; Li et al., 2024; Khreish et al., 2024). Despite their established clinical relevance, prior studies have predominantly employed cross-sectional designs and subjective, semi-quantitative grading—limiting longitudinal insight, inter-rater reliability, and clinical scalability. Critically, robust longitudinal quantification of FTD and PPA dynamics in children remains lacking, and the precise axial length–associated inflection point at which these parameters shift from adaptive to maladaptive trajectories has yet to be empirically established (Cheng et al., 2021; Yan et al., 2015).

Pathological basis of myopic posterior-pole remodelling. Myopic axial elongation is not passive stretching but an active, cell-mediated remodelling of the posterior ocular coats. In the growing sclera, sustained mechanical signalling shifts fibroblast phenotype towards a catabolic state, with upregulation of matrix metalloproteinases, loss of proteoglycans, reduced collagen fibril diameter and diminished cross-linking; the net result is thinning of the posterior sclera, reduced elastic modulus and increased viscoelastic creep, so that a given tensile load produces progressively greater deformation (Rada et al., 2006; McBrien et al., 2009; Boote et al., 2020). Because the paediatric sclera remains in an active developmental phase with high extracellular-matrix plasticity and lower effective stiffness than the mature eye, its threshold for biomechanical decompensation is expected to be reached at a smaller absolute axial dimension than in adults. Immediately internal to the sclera, the choroid acts as a bidirectional, rapidly modifiable buffer—thickening during emmetropization and thinning as elongation outpaces its capacity, with choriocapillaris compression and stromal atrophy progressively unmasking large choroidal vessels, the histological substrate of fundus tessellation (Xue et al., 2024; Zhou et al., 2018; Jin et al., 2019; Fontaine et al., 2017; Read et al., 2015; Venkatesh et al., 2026). At the optic nerve head, elongation drives displacement of the RPE–Bruch’s membrane complex, generating peripapillary atrophy as a marker of accrued scleral strain (Lee et al., 2018; Kim et al., 2018; Jonas et al., 2017; Wang et al., 2021). These distinct tissue processes—choroidal thinning versus peripapillary displacement—suggest that FTD and PPA may capture different aspects of posterior-pole remodelling, and that their relationship with AL may not be linear. However, the precise shape of these relationships and the AL value at which adaptive remodelling transitions to maladaptive change remain to be empirically determined in children.

In recent years, advances in artificial intelligence (AI) and deep learning have enabled transformative progress in the quantitative analysis of ophthalmic imaging data. By leveraging state-of-the-art architectures—including convolutional neural networks (CNNs) and vision Transformers—AI systems now convert qualitative, observer-dependent morphological assessments into objective, continuous, and highly reproducible quantitative metrics (Li et al., 2022; Li et al., 2023; Xu and Yang, 2023; Wu et al., 2024). This capability facilitates precise longitudinal tracking of subtle structural evolution in the fundus during axial elongation and supports the data-driven identification of biologically meaningful inflection points that demarcate the transition from physiological adaptation to pathological remodeling. Notably, while an AL exceeding 26 mm is widely adopted as a clinical benchmark for high myopia and associated complications in adults, no rigorously validated, age-specific AL threshold currently exists to define the onset of pathological remodeling in school-aged children (Wang et al., 2023; Haarman et al., 2020). Pediatric eyes exhibit greater scleral extensibility and reduced tissue stiffness compared with adult eyes, rendering them more susceptible to biomechanical decompensation at lower AL values. Consequently, establishing sensitive, imaging-based biomarkers that directly reflect the structural integrity of the posterior pole—specifically the retina–RPE–choroid complex—and empirically determining the critical AL at which this complex shifts from adaptive homeostasis to maladaptive remodeling are essential for enabling precision-targeted, early-stage interventions in pediatric myopia management.

Based on two consecutive years of longitudinal ophthalmic screening data and state-of-the-art artificial intelligence–driven quantitative imaging, this study aims to (Holden et al., 2016): characterize the longitudinal trajectories of FTD and PPA area during myopia progression in adolescents (Vagge et al., 2026); identify data-driven subtypes of myopia progression through unsupervised clustering of AL growth velocity, and assess inter-subtype heterogeneity in posterior pole structural remodeling; and (Liu et al., 2026) empirically determine a biologically informed, nonlinear transition threshold for AL—defined as the inflection point at which FTD and PPA dynamics shift from adaptive stabilization to pathological acceleration—using segmented regression modeling across the entire cohort.

## 2 Materials and methods

### 2.1 Study design and participants

**FIGURE 1:**
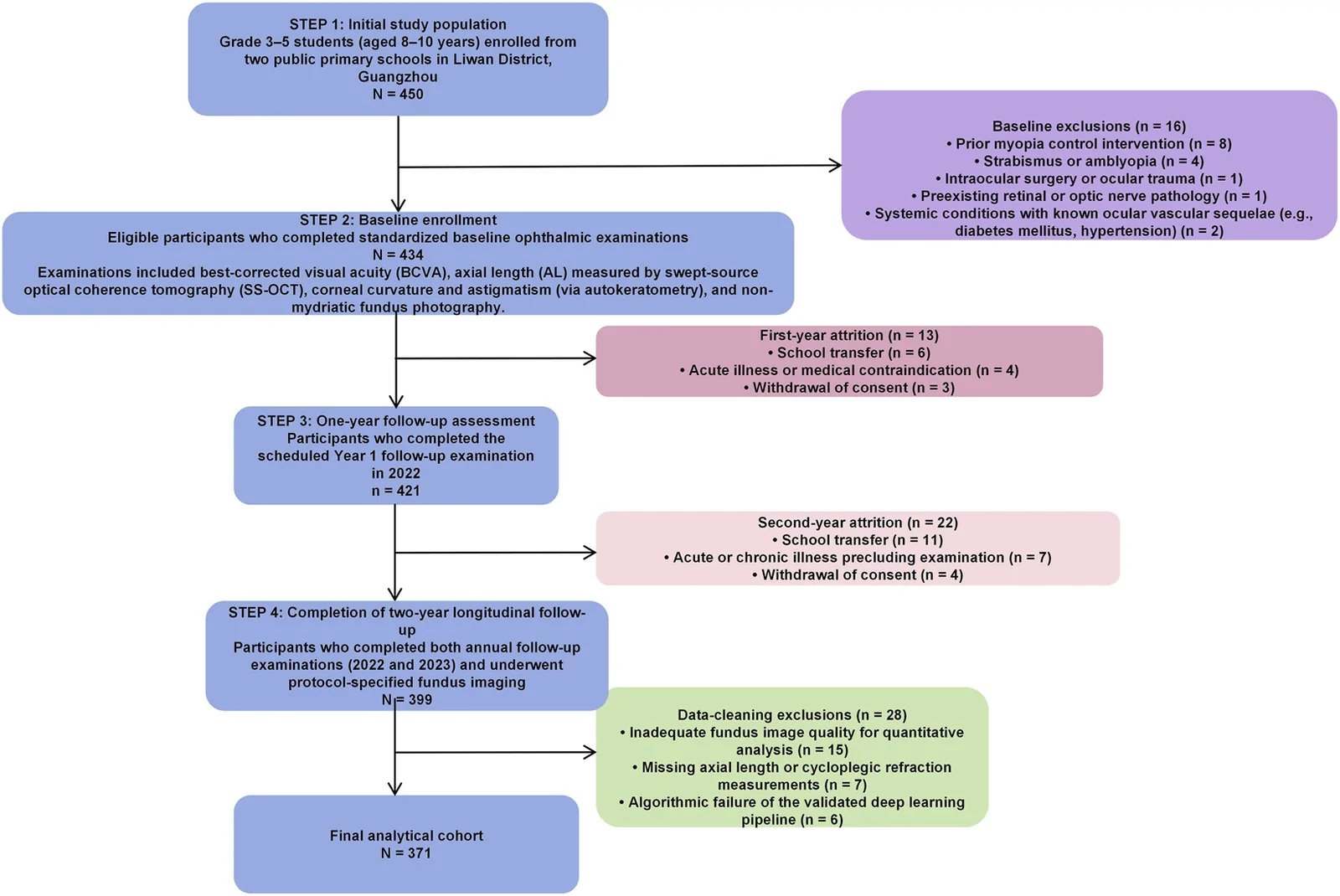
Participant enrollment, screening, and longitudinal follow-up flowchart.

This study employed a prospective longitudinal cohort design. Participants were recruited from Grades 3 to 5 at two primary schools in Liwan District, Guangzhou, and underwent annual ophthalmic examinations from 2021 (baseline) through 2023. The study protocol was approved by the Ethics Committee of Guangzhou First People’s Hospital (approval number: K-2024-032-02) and conducted in full accordance with the principles of the Declaration of Helsinki. Written informed consent was obtained from both participants and their legal guardians prior to enrollment. Inclusion criteria specified age 8–11 years at baseline and completion of two consecutive annual follow-up visits. Exclusion criteria comprised (Holden et al., 2016): receipt of any myopia control intervention (e.g., orthokeratology, low-dose atropine, or multifocal contact lenses) (Vagge et al., 2026); history of strabismus, amblyopia, intraocular surgery, or ocular trauma (Liu et al., 2026); media opacities precluding high-fidelity fundus imaging—including corneal scarring, cataract, or vitreous haze (Yamashita et al., 2024); pre-existing retinal or optic nerve pathology (e.g., glaucoma, retinitis pigmentosa, uveitis, or optic neuropathy) (Jiang et al., 2024); systemic vascular disorders known to affect ocular perfusion (e.g., diabetes mellitus or hypertension); and (Lim et al., 2025) fundus images failing predefined quality standards for artificial intelligence–based quantitative analysis—specifically, pupil diameter <3 mm, significant motion artifact, inadequate focus, or suboptimal illumination. The standardized examination workflow is illustrated in Figure 1. Uncorrected distance visual acuity (UDVA) was assessed using a back-illuminated logarithmic ETDRS chart under photopic conditions. Ocular biometry—including AL and corneal radius of curvature—was performed using a validated optical biometer (Nidek AL-Scan), with the mean of three repeated measurements used for analysis. For fundus imaging, 50° color fundus photographs centered on both the macula and optic disc were acquired using a fully automated non-mydriatic retinal camera (Reticam 3,100, New Vision Opto-Electronic Co., Ltd., Chongqing, China) in a dimly lit room. Image eligibility for AI quantification required clear visualization of retinal vasculature, optic disc margins, and foveal landmarks—ensuring anatomical fidelity for downstream FTD and PPA measurement.

### 2.2 Image analysis and quantitative parameters

#### 2.2.1 Image processing

**FIGURE 2:**
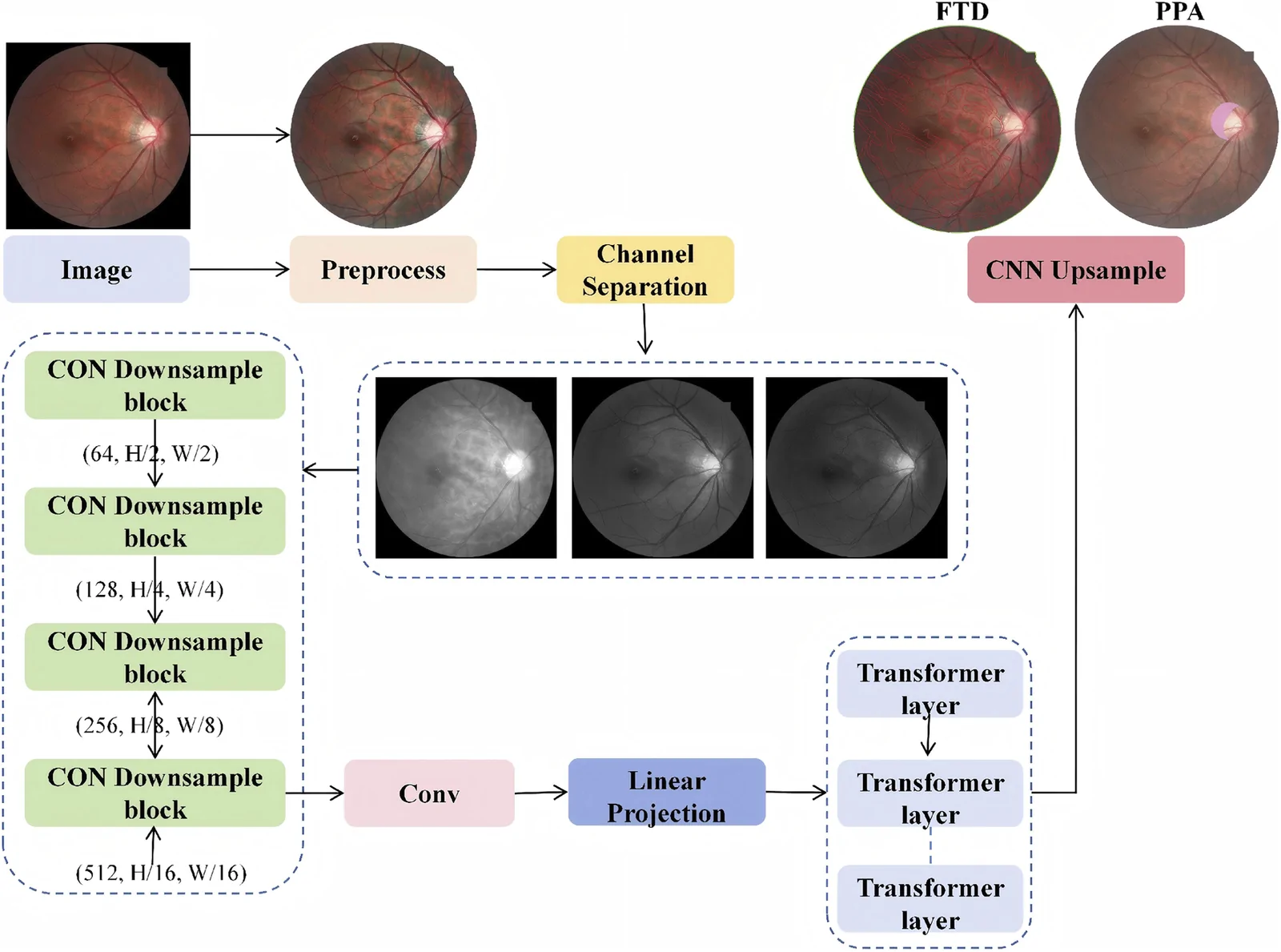
AI-based workflow for fundus image quantification of FTD and PPA. Representative quantitative outputs are shown at the top right: the FTD region is indicated by the red contour, and the total PPA region is indicated in pink.

All fundus images were processed using a validated deep learning–based quantitative analysis platform (EVisionAI, EVision Technology (Beijing) Co., Ltd., China) (He et al., 2024); the workflow is summarised in Figure 2. Preprocessing comprised automated extraction of the central fundus region of interest, conversion to the CIELAB colour space with intensity normalisation to mitigate inter-device and inter-session illumination variability, and contrast-limited adaptive histogram equalisation (CLAHE) to enhance microstructural contrast at tissue interfaces critical for posterior-pole assessment (Shao et al., 2021; Wan et al., 2021). The normalised image was then separated into its constituent colour channels, which were processed in parallel as single-channel inputs. A TransUNet-style hybrid convolutional–Transformer network was used for segmentation: four successive convolutional down-sampling blocks generated feature maps of 64, 128, 256 and 512 channels at 1/2 to 1/16 of the input resolution; the deepest features were linearly projected into token embeddings and processed by stacked Transformer layers to model global context and long-range spatial dependencies; and full spatial resolution was restored through a convolutional up-sampling decoder. The network output pixel-level maps of the peripapillary atrophic region and of tessellated (exposed large choroidal vessel) pixels, identified from en-face colour, reflectance and texture characteristics with the automatically detected optic disc margin and foveal centre as anatomical references. Total PPA area and FTD were derived from these maps (Figure 2; FTD, red contour; total PPA, pink region). The platform has previously demonstrated high intra- and inter-session repeatability and strong agreement with expert grading (He et al., 2024; Shao et al., 2021; Huang et al., 2025).

Model development and validation. The segmentation models within the platform were developed and validated by the manufacturer on large-scale, expert-annotated multicentre Chinese datasets, comprising 40,600 fundus photographs from a Shanghai community-based cohort, 12,067 photographs from 5,320 patients at Beijing Tongren Hospital, and 2,981 patients from Tianjin Eye Hospital, all acquired with standardised non-mydriatic fundus cameras (He et al., 2024; Long et al., 2022; Zou et al., 2025). Ground truth was established pixel-wise by board-certified ophthalmologists using a machine-assisted annotation workflow, with a senior retinal specialist serving as the final adjudicator (He H et al., 2023). On independent test sets, segmentation performance was as follows: PPA—accuracy 0.999, sensitivity 0.968, specificity 0.999, IoU 0.894; optic disc—accuracy 0.999, sensitivity 0.992, IoU 0.957 (He et al., 2024); and fundus tessellation detection—accuracy 0.9766, sensitivity 0.9551, specificity 0.9801 (He X et al., 2023). Pixel-to-millimetre calibration was validated against the ISO 10940:2009 standard across four camera models, with <5% bias relative to manual ISO measurement and a coefficient of variation for repeated ROI diameter measurement of 2.85 × 10^−4^ (Canon) and 1.96 × 10^−3^ (Topcon) (Long et al., 2022). No retraining or fine-tuning was performed for the present study; the pretrained model was applied unchanged, and its agreement with expert grading in this cohort was verified as described in Section 2.2.3. Representative de-identified fundus photographs with AI segmentation overlays (FTD and PPA) are provided in Supplementary Figure S1 to illustrate the model output quality lesions.

#### 2.2.2 Quantitative parameters

FTD: FTD was defined as the proportion of the photographed fundus area occupied by fundus tessellation:

$$\text{FTD}={\text{Area}}_{\text{tessellation}}/\;{\text{Area}}_{\text{ROI}}$$

The segmentation network generated two masks from each colour fundus photograph: the tessellated region, defined as areas in which large choroidal vessels were visible through an attenuated retinal pigment epithelium, and the region of interest corresponding to the entire photographed fundus area. FTD was computed as the ratio of the pixel count within the tessellated mask to that within the ROI mask, expressed as a percentage (He et al., 2024; Huang et al., 2025). Because FTD is a dimensionless area ratio obtained from images acquired with the same camera, field of view and acquisition protocol at every visit, the denominator remained constant across visits, and the measure is inherently independent of changes in retinal image magnification associated with axial elongation.

PPA area: PPA was quantified on 50° optic disc-centered color fundus photographs as the total area of the peripapillary atrophic crescent. The segmentation model identified an inner boundary contiguous with the outer optic disc margin and an outer boundary where the atrophic appearance ended and normal fundus reflectance resumed. The enclosed pixel area was converted to mm^2^ using device-specific spatial calibration:

$$\text{Area }\left({\text{mm}}^{2}\right)=\text{Area }\left(\text{pixels}\right)\times d^{2}\text{resolution }/\;10^{6}$$

where *d*_resolution_ is in μm/pixel.

All FTD and PPA measurements were obtained using the identical software version and parameter settings at baseline and at each annual follow-up visit, yielding one value per eye per visit; these repeated measurements formed the longitudinal dataset analysed as described in Section 2.3.

#### 2.2.3 Quality control

All ophthalmic instruments underwent scheduled calibration according to manufacturer specifications prior to each examination session. Image analysis was conducted using a standardized, version-controlled AI algorithm; analysts were blinded to participant identifiers and all clinical metadata throughout the analysis process. To assess analytical validity, a random 10% sample of fundus images was independently reviewed by two board-certified senior ophthalmologists for segmentation accuracy—specifically evaluating delineation of the optic disc margin, retinal vasculature, and PPA boundaries. Inter-rater agreement between AI outputs and expert consensus was quantified using the two-way random-effects intraclass correlation coefficient (ICC, absolute agreement definition). All reported parameters achieved ICC values ≥ 0.92, confirming excellent reliability of AI-derived quantitative measurements relative to expert grading.

### 2.3 Statistical analysis

AI-quantified FTD and PPA values from each visit (Figure 2) were analysed longitudinally as follows. Statistical analyses were conducted using SPSS 27.0 (IBM Corp., Armonk, NY, United States) and Python 3.9 (SciPy, statsmodels, and scikit-learn libraries). Two-sided *P* < 0.05 was considered statistically significant. Annualized rates of change for each ocular parameter were estimated using ordinary least squares (OLS) linear regression on individual longitudinal trajectories. Myopia progression subtypes were identified via k-means clustering applied to annual AL elongation rates. Linear mixed-effects models (LMMs), with random intercepts and slopes for participant ID, were fitted to assess the longitudinal associations between AL and fundus structural parameters—accounting for within-subject correlation and variable follow-up intervals. Pearson correlation coefficients and univariate linear regression were used to quantify pairwise relationships among parameter growth rates. The critical AL inflection point—defined as the AL value at which the quadratic term in the AL–fundus parameter relationship becomes statistically significant—was determined using segmented quadratic regression with 1,000-bootstrap resampling to estimate 95% confidence intervals. To visualize AL-dependent transitions, we generated continuous first-derivative curves for baseline FTD and PPA (AL 22–26 mm) from quadratic and spline models, with 95% bootstrap confidence bands (1,000 participant-level resamples). Sparse-data regions beyond the 5th–95th AL percentiles were marked as extrapolation zones. Sex-stratified nonlinear analyses were prespecified as secondary analyses. In the full cohort, sex modification of the AL–biomarker association was tested by adding sex interaction terms with both the linear and quadratic AL terms: biomarker ∼ AL + AL^2^ + Sex + AL × Sex + AL^2^ × Sex + Age + (1 + AL | ID). The joint contribution of AL × Sex and AL^2^ × Sex was evaluated using likelihood-ratio tests (2 df). We then refitted sex-stratified quadratic mixed models and estimated turning points as AL* = −*β*1/(2*β*2), with participant-level cluster bootstrap (resampling IDs within sex) used to obtain percentile confidence intervals for sex-specific turning points and their between-sex difference. To contextualize the 24.0-mm candidate transition zone, we mapped AL = 24.0 mm to age- and sex-specific pediatric percentiles using published Chinese normative AL data (numerical percentile tables) (He X. et al., 2023). Percentile positions were estimated by piecewise linear interpolation between adjacent percentile knots (*P*_3_, *P*_5_ *, P*_10_ *,P*_25_ *, P*_50_ *, P*_75_ *, P*_90_ *, P*_95_). We then compared these normative positions with the visit-level distribution of AL in our cohort (Supplementary Table S1; Supplementary Figure S2).

## 3 Results

### 3.1 Baseline characteristics and overall longitudinal trends

**FIGURE 3:**
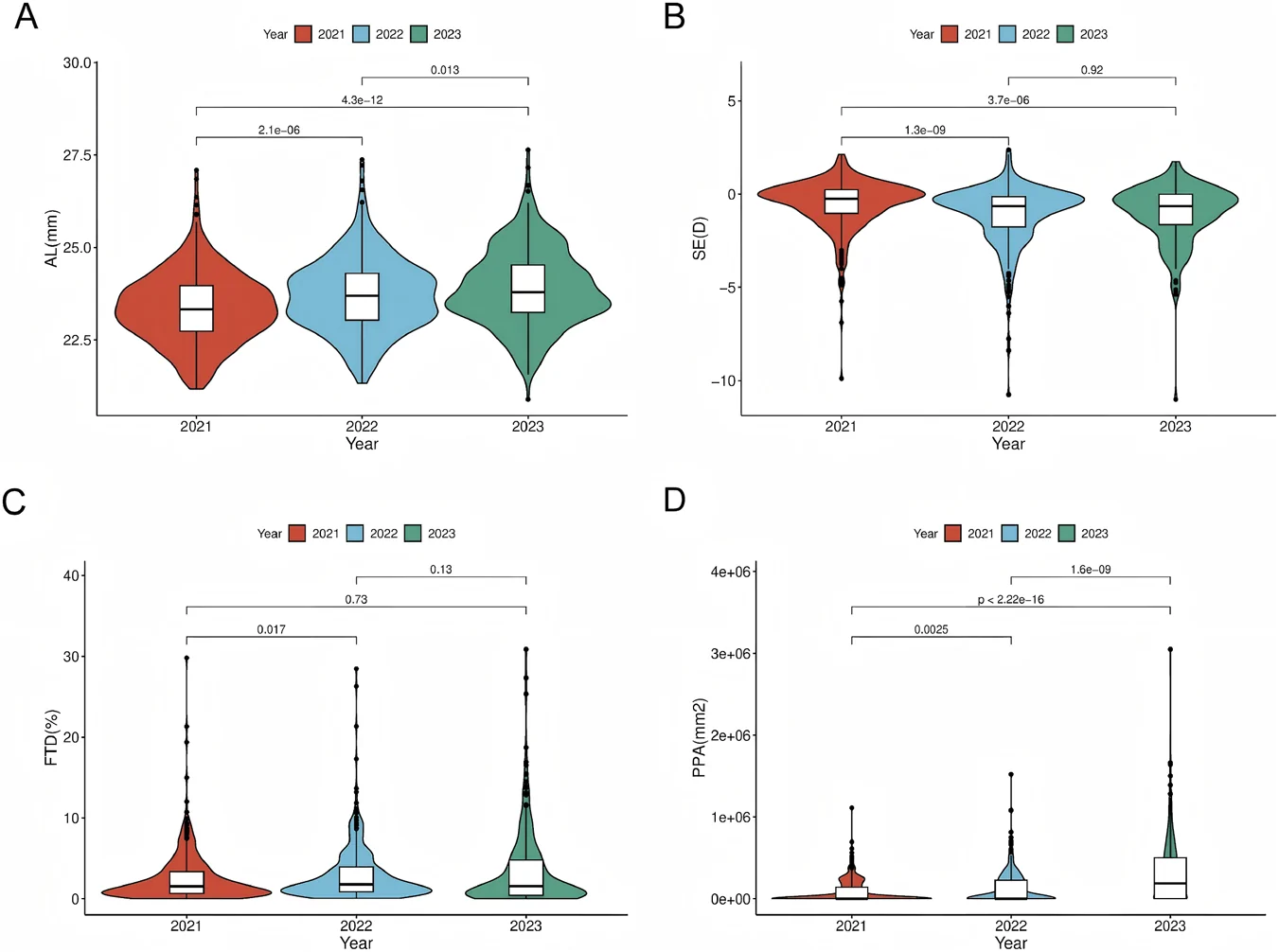
Annualized changes in ocular biometric and fundus structural parameters across 2 years of follow-up. **(A)** Axial length (AL); **(B)** spherical equivalent (SE); **(C)** fundus tessellation density (FTD); **(D)** peripapillary atrophy (PPA) area. AL, PPA, and FTD (in Year 1) increased significantly over time (all *p* < 0.001, linear mixed-effects models), whereas SE exhibited progressive myopic drift (*p* < 0.001). Horizontal brackets denote pairwise intergroup comparisons; Bonferroni-corrected p-values are annotated above each bracket. Violin plots display kernel density estimates; embedded box plots indicate interquartile range (IQR), with medians shown as central horizontal lines. Data are presented as group-specific mean values with error bars indicating 95% confidence intervals (CIs) estimated from linear mixed-effects models.

A total of 371 children were enrolled in this study (right-eye data only), including 131 in the stable progression group, 157 in the moderate progression group, and 83 in the rapid progression group. The demographic and ocular baseline characteristics of the cohort are summarized in Table 1. The median age was 9.00 years (IQR, 9.00–10.00), and 202 participants (54.4%) were male. The median SE was −0.13 D (IQR, −1.13 to 0.25), the median AL was 23.29 mm (IQR, 22.70–23.94), the mean corneal radius of curvature (CR) was 7.80 ± 0.26 mm, and the median AL/CR ratio was 2.98 (IQR, 2.92–3.05). Across myopia progression phenotypes, baseline sex distribution (*P* < 0.001) and AL/CR ratio (*P* = 0.021) differed significantly, whereas age, SE, AL, and CR were comparable among groups (all *P* > 0.05). Over the 2-year period, AL increased progressively (Figure 3A), with the violin plot revealing an upward shift in its distribution and a right-skewed tail—indicative of substantial inter-individual heterogeneity in elongation velocity, including a subset exhibiting accelerated growth. SE also demonstrated progressive myopization and declined significantly each year (all *p* < 0.001), confirming sustained refractive deterioration (Figure 3B). FTD increased significantly from baseline to Year 1 (*p* = 0.017), reflecting early structural remodeling of the posterior pole; however, no further significant change occurred between Year 1 and Year 2 (*p* = 0.73). This plateau at the cohort level obscures subtype-specific dynamics—as subsequently revealed by progression phenotyping (Section 3.4). Similarly, PPA area expanded annually (Figure 3D), consistent with cumulative biomechanical stress at the peripapillary sclera during axial elongation.

**TABLE 1:** Baseline characteristics of the study cohort, overall and stratified by myopia progression phenotype.

Characteristic	Overall (n = 371)	Stable progression (n = 131)	Moderate progression (n = 157)	Rapid progression (n = 83)	P Value
Age at baseline, years	9.00 [9.00, 10.00]	9.00 [9.00, 10.00]	9.00 [9.00, 10.00]	9.00 [9.00, 10.00]	0.745
Male sex, n (%)	202 (54.4)	82 (62.6)	91 (58.0)	29 (34.9)	<0.001
Spherical equivalent, D	−0.13 [-1.13, 0.25]	−0.25 [-1.13, 0.38]	−0.13 [-1.00, 0.25]	−0.38 [-1.06, 0.13]	0.430
Axial length, mm	23.29 [22.70, 23.94]	23.34 [22.80, 24.02]	23.23 [22.69, 23.87]	23.38 [22.65, 23.91]	0.539
Corneal radius of curvature, mm	7.80 (0.26)	7.85 (0.25)	7.79 (0.27)	7.78 (0.25)	0.081
AL/CR ratio	2.98 [2.92, 3.05]	2.96 [2.92, 3.02]	2.98 [2.92, 3.06]	3.00 [2.95, 3.06]	0.021

### 3.2 Analysis of myopia progression subtypes based on axial length growth velocity

#### 3.2.1 Unsupervised clustering and validation of longitudinal axial length trajectories

**FIGURE 4:**
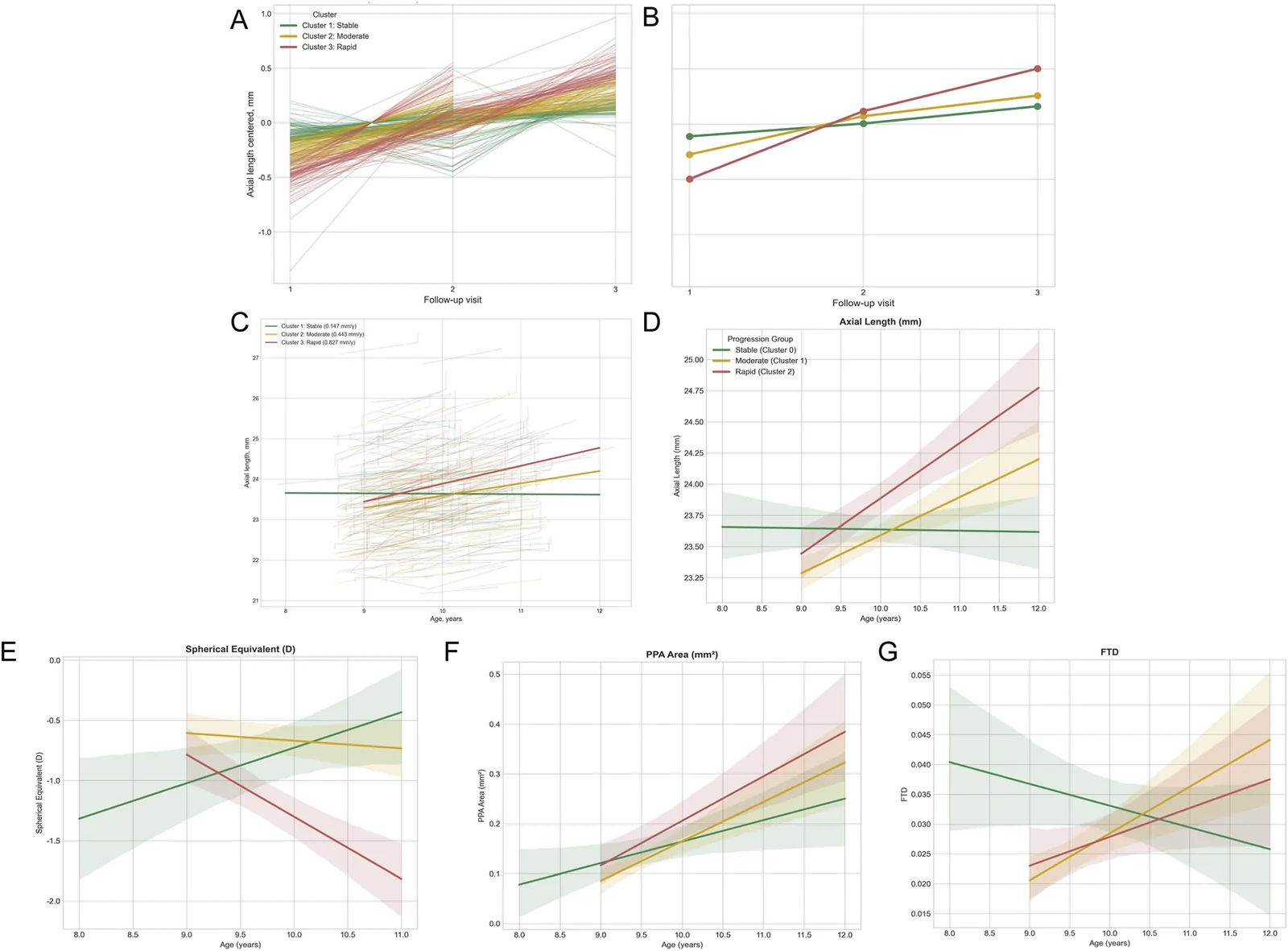
Axial length–based clustering and longitudinal trajectories of ocular parameters across myopia progression subtypes (stable, n = 131; moderate, n = 157; rapid, n = 83; total n = 371 children). **(A)** Individual-centered axial length (AL) trajectories aligned to baseline age, stratified into stable, moderate, and rapid progression groups using K-means clustering based on annual AL elongation rate. **(B)** Mean ± SEM AL trajectories for each subgroup, showing distinct growth patterns, with the rapid progression group exhibiting the steepest elongation. **(C)** AL versus age with group-specific ordinary least-squares fits (stable: slope = 0.147 mm/y; moderate: slope = 0.443 mm/y; rapid: slope = 0.827 mm/y), all with high *R* ^2^ values (>0.97), indicating strong linear associations. **(D–G)** Age-dependent longitudinal changes in ocular parameters across progression subtypes: **(D)** AL, **(E)** spherical equivalent (SE), **(F)** peripapillary atrophy area (PPA), and **(G)** fundus tessellation density (FTD). Baseline-level (intercept) differences: rapid vs. stable, *β* = 0.400 mm (95% CI 0.148 to 0.652; *p* = 0.002); moderate vs. stable, *β* = 0.129 mm (95% CI −0.031 to 0.289; *p* = 0.113). Shaded areas represent 95% confidence intervals derived from linear mixed-effects models. The rapid progression group demonstrates accelerated AL elongation and PPA expansion, a greater myopic shift in SE, and a distinct nonlinear trajectory in FTD.

To characterize inter-individual heterogeneity in myopia progression, participants were stratified into three data-driven subtypes using k-means clustering applied to annual AL elongation rates (Figures 4A–C). Cluster validity was confirmed via silhouette analysis (mean silhouette width = 0.68) and Calinski–Harabasz index maximization. The resulting subtypes were (Holden et al., 2016): Stable: mean AL elongation rate = 0.15 mm/year—consistent with age-expected physiological growth and minimal posterior pole remodeling (Vagge et al., 2026); Moderate: 0.44 mm/year—representing typical progressive myopia; and (Liu et al., 2026) Rapid: 0.83 mm/year—indicating accelerated axial elongation associated with elevated risk of early fundus pathology. Figures 4A–C displays individual AL trajectories and group-level mean curves, demonstrating statistically distinct longitudinal patterns (*p* < 0.001 for between-group slope differences, linear mixed-effects model). Linear regression slopes for each group aligned closely with observed growth rates (*R* ^2^ > 0.97), confirming the robustness and biological plausibility of the clustering solution.

To rigorously evaluate inter-subtype differences in AL trajectories, linear mixed-effects models (LMMs) were fitted with random intercepts and slopes for participant ID, adjusting for baseline age and sex. Results revealed that, after covariate adjustment, the rapid progression group exhibited a significantly higher baseline AL elongation rate compared with the stable group (*β* = 0.400 mm/year; 95% CI: 0.148–0.652; *p* = 0.002). In contrast, no statistically significant difference was observed between the moderate and stable groups (*β* = 0.129 mm/year; 95% CI: −0.031 to 0.289; *p* = 0.113). Longitudinally, AL increased significantly with age across the cohort (*β* = 0.164 mm/year; *p* < 0.001). Relative to the stable group, both the moderate (*β* = 0.277 mm/year; *p* < 0.001) and rapid (*β* = 0.653 mm/year; *p* < 0.001) groups demonstrated significantly accelerated annual AL elongation. Collectively, these findings confirm distinct AL developmental trajectories across subtypes—most notably, the rapid progression phenotype is characterized by both elevated AL and markedly accelerated growth velocity, consistent with early pathological myopia.

#### 3.2.2 Longitudinal parameter trajectories across myopia progression subtypes

To determine whether accelerated AL elongation is associated with differential fundus microstructural remodeling, we examined longitudinal trajectories of PPA area and FTD across the three progression subtypes defined by annual AL growth velocity. Figures 4D–G presents age-adjusted trajectories of AL, spherical equivalent (SE), PPA area, and FTD for each subgroup. All groups exhibited linear AL increase with age; however, the slope differed significantly: steepest in the rapid group (0.83 mm/year), intermediate in the moderate group (0.44 mm/year), and shallowest in the stable group (0.15 mm/year) (*p* < 0.001 for between-group slope differences, LMM). SE declined progressively in all subtypes, with the most pronounced myopic shift observed in the rapid group (mean annual change: −0.72 D vs. −0.31 D in stable group; *p* < 0.001). PPA area expanded annually in all groups, but the magnitude followed a clear inter-subtype gradient: 0.242 mm^2^/year in the rapid group, 0.139 mm^2^/year in the moderate group, and 0.076 mm^2^/year in the stable group (*p* < 0.001 for trend across ordered groups, Cochran–Armitage test). This monotonic association supports a dose–response relationship between AL elongation velocity and peripapillary scleral remodeling. In contrast, FTD changes were modest overall but exhibited subtype-specific dynamics: annual increases were 0.004, 0.005, and 0.006 units/year in the stable, moderate, and rapid groups, respectively (*p* = 0.042 for linear trend). Notably, while absolute changes were small, the progressive rise across subtypes—particularly the divergence emerging after Year 1—suggests that FTD may serve as a sensitive, stage-dependent biomarker of posterior pole biomechanical adaptation.

#### 3.2.3 Correlation analysis of longitudinal parameter changes across myopia progression subtypes

**FIGURE 5:**
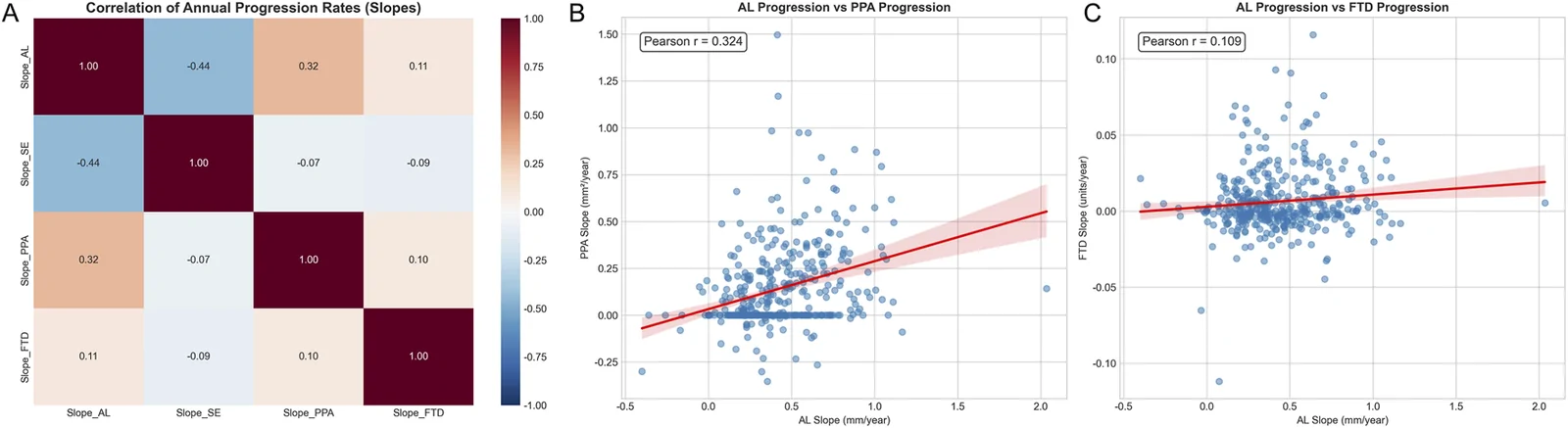
Correlation structure of annualized change rates (individual-level slopes from linear mixed-effects models; n = 371 children). **(A)** Pearson correlation matrix for annualized change in AL (mm/y), SE (D/y), PPA (mm^2^/y), and FTD (%/y). **(B)** ΔAL versus ΔPPA: r = 0.324, *p* < 0.001; regression slope = 0.287 mm^2^ per mm AL increase, *R* ^2^ = 0.105; n = 371. **(C)** ΔAL versus ΔFTD: r = 0.109, *p* = 0.120; slope = 1.10% per mm AL increase, *R* ^2^ = 0.012; n = 371. Solid lines represent ordinary least-squares (OLS) fits; shaded areas indicate 95% confidence intervals. Each point represents one participant. Statistical significance was set at *p* < 0.05; all *p*-values are two-sided.

Within each progression subtype, annualized rates of change were computed for AL, spherical equivalent (SE), PPA area, and FTD. Pairwise associations among these rates were assessed using Pearson correlation coefficients and univariate linear regression. The correlation heatmap (Figure 5A) revealed a moderate positive association between AL elongation rate and PPA expansion rate (r = 0.324, *p* < 0.001), consistent with biomechanical strain–mediated scleral remodeling at the optic nerve head. In contrast, the correlation between AL elongation rate and FTD change rate was weak and nonsignificant (r = 0.109, *p* = 0.12), reflecting the underlying U-shaped, nonlinear relationship between FTD and AL—characterized by initial physiological decline followed by pathological rebound—as previously established in Section 3.4. Scatter plots with superimposed linear regression lines (Figures 5B,C) corroborated these findings: AL versus PPA exhibited a clear positive linear trend (slope = 0.287 mm^2^/mm AL increase; *R* ^2^ = 0.105), whereas AL versus FTD showed minimal slope (0.011 units/mm) and negligible explanatory power (*R* ^2^ = 0.012). Collectively, these analyses confirm that PPA expansion is robustly coupled to AL elongation velocity, while FTD dynamics are governed primarily by nonlinear, threshold-dependent mechanisms rather than linear growth kinetics.

### 3.3 Longitudinal association between axial length elongation and fundus structural parameters

To quantify the longitudinal association of AL with fundus structural biomarkers, linear mixed-effects models (LMMs) were fitted with AL as the primary fixed-effect predictor, adjusting for age and sex as covariates. The model specification was:

$$\text{Biomarker }∼\text{ AL}+\text{Age}+\text{Gender}+\left(\left.1\right|\text{ID}\right)$$

As shown in Table 2, after multivariable adjustment, AL exhibited a statistically significant positive association with PPA area (*β* = 0.127 mm^2^/mm; 95% CI: 0.098–0.156; *p* < 0.001): each 1-mm increase in AL corresponded to an average 0.127-mm^2^ expansion in PPA. This robust, dose-dependent relationship supports PPA as a quantitative biomechanical indicator of scleral deformation at the optic nerve head—directly reflecting cumulative mechanical strain on peripapillary tissues. Similarly, Table 2 reports a statistically significant but quantitatively modest association between AL and FTD (*β* = 0.011 units/mm; 95% CI: 0.008–0.014; *p* < 0.001). While this linear coefficient is small, it represents the net population-level trend across the full AL range. As established in Section 3.4, the underlying AL–FTD relationship is intrinsically nonlinear (U-shaped), with opposing directional effects below and above the inflection threshold (∼23.66 mm). Consequently, the small positive *β* reflects the dominance of the pathological upward phase in the overall cohort distribution—not a uniform linear response.

**TABLE 2:** Linear mixed - effects models for evaluating the associations between axial length (AL) and ocular parameters (PPA and FTD).

Ocular parameter	Parameter	Coef	Std.Err	z	P > |z|	[0.025	0.975]
PPA (mm^2^)	Intercept	−3.507	0.235	−14.897	0.000 ***	−3.968	−3.046
	AL	0.127	0.011	11.805	0.000 ***	0.106	0.148
	Group Var	0.032	0.023	​	​	​	​
FTD	Intercept	−0.239	0.031	−7.759	0.000 ***	−0.299	−0.178
	AL	0.011	0.001	7.401	0.000 ***	0.008	0.014
	Group Var	0.001	0.005	​	​	​	​

PPA, is expressed in mm^2^; AL, is expressed in mm. *** indicates *p* < 0.001.

### 3.4 Model selection across functional forms (AIC/BIC-based)

To evaluate which nonlinear specification was most appropriate for the derivative analyses, we compared quadratic, piecewise linear (segmented), and natural cubic spline models for each AL-related outcome (all adjusted for baseline age; Supplementary Table S2). Among these nonlinear forms, the quadratic model consistently performed best for Baseline FTD, Baseline PPA, and Slope FTD, with the lowest AIC and BIC within the nonlinear set (Supplementary Table S2).

For Baseline FTD, the quadratic model (AIC = −357.58, BIC = −345.48) outperformed segmented (AIC = −356.03, BIC = −341.51) and spline models (AIC = −355.82, BIC = −341.30), with ΔAIC of 1.55 and 1.76, respectively, and ΔBIC of 3.97 and 4.18 against spline. For Baseline PPA, the quadratic model (AIC = −117.07, BIC = −104.98) was superior to segmented (AIC = −116.40, BIC = −101.89) and spline (AIC = −115.45, BIC = −100.94), with ΔAIC = 0.67 and 1.62, respectively, and ΔBIC against spline = 4.04. For Slope FTD, the quadratic model (AIC = −426.38, BIC = −414.28) again outperformed segmented (AIC = −425.68, BIC = −411.16) and spline (AIC = −424.50, BIC = −409.98), with ΔAIC = 0.70 and 1.88, respectively. For Slope PPA, the spline model had the marginally smallest AIC (AIC = 4.15), but the difference from the quadratic was negligible (ΔAIC = 0.10), and BIC penalized the spline complexity (BIC: quadratic 17.13, spline 18.66, ΔBIC = 1.53 in favor of quadratic).

**FIGURE 6:**
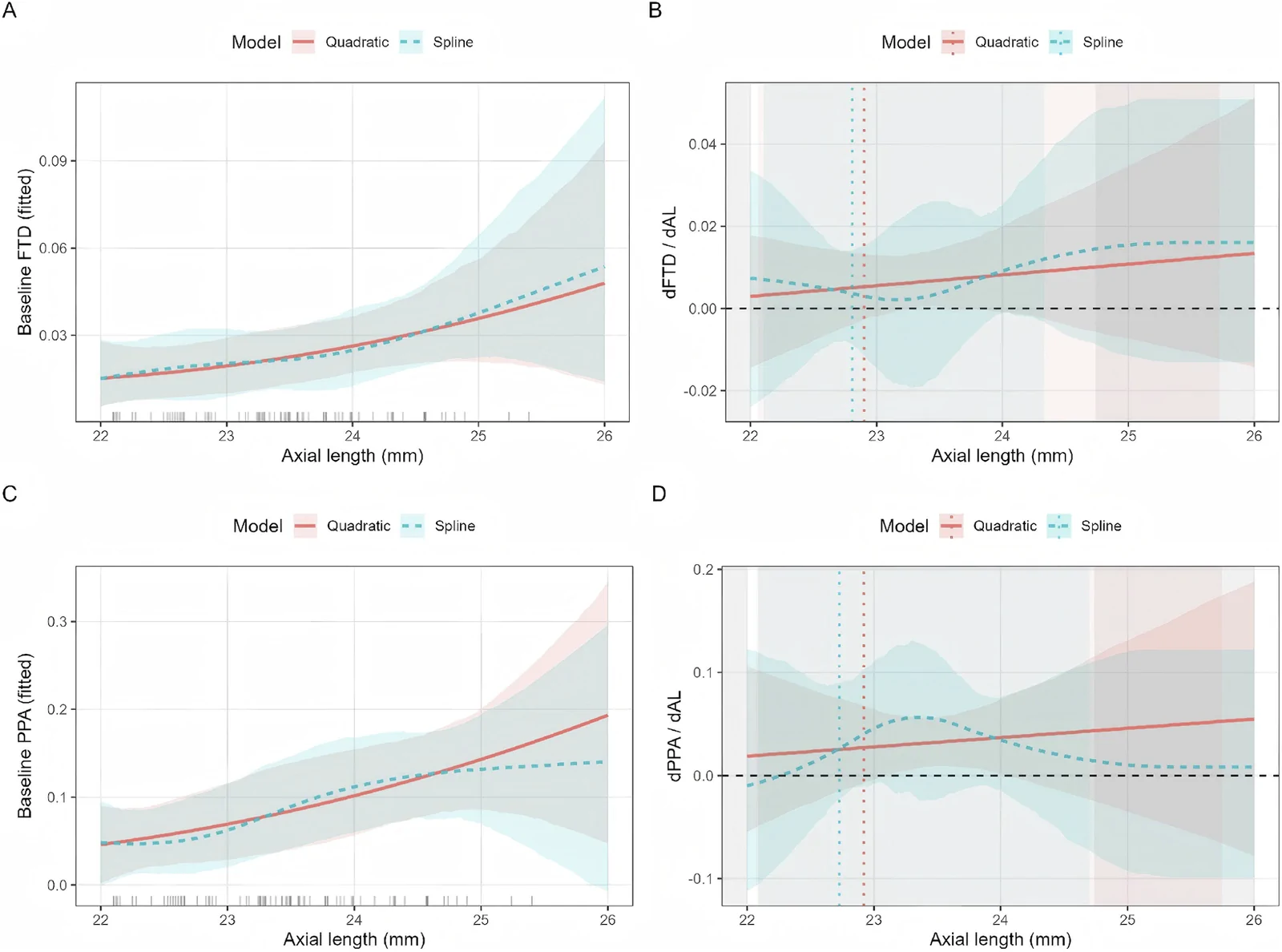
Continuous fitted and first-derivative curves of baseline fundus biomarkers across axial length (AL, 22–26 mm). **(A)** Baseline FTD fitted against AL; **(B)** first derivative of FTD with respect to AL (dFTD/dAL); **(C)** baseline PPA fitted against AL; **(D)** first derivative of PPA with respect to AL (dPPA/dAL). Quadratic and natural cubic spline models are shown with bootstrap-derived 95% confidence bands (1,000 resamples). Vertical dotted lines indicate model-specific median derivative zero-crossing locations (FTD: 22.90 mm [quadratic], 22.81 mm [spline]; PPA: 22.92 mm [quadratic], 22.73 mm [spline]). Shaded vertical bands denote corresponding 95% confidence intervals of zero-crossings. Gray side regions mark sparse-data/extrapolation areas outside the empirical AL 5th–95th percentile support (21.767–24.737 mm).

Segmented regression did not show statistically significant slope-change evidence in any outcome (Davies test: Baseline FTD *P* = 1.000, Baseline PPA *P* = 0.951, Slope FTD *P* = 0.185, Slope PPA *P* = 0.334). Accordingly, we selected the quadratic model for the primary derivative visualization (Figure 6) due to its consistent performance among nonlinear forms, while acknowledging that precise turning-point localization remains exploratory in this summary-data analysis.

### 3.5 Continuous first-derivative curves of AL-related change

Continuous first-derivative analyses of baseline FTD and baseline PPA across the AL range of 22–26 mm are presented in Figure 6.

Bootstrap-based zero-crossing summaries indicated median derivative transition locations at 22.90 mm for FTD in the quadratic model (95% CI: 22.06–25.72) and 22.81 mm in the spline model (95% CI: 22.11–24.33). For PPA, the corresponding median transition locations were 22.92 mm in the quadratic model (95% CI: 22.07–25.74) and 22.73 mm in the spline model (95% CI: 22.09–24.70).

The empirical AL support in this dataset was 21.767–24.737 mm (5th–95th percentiles). Accordingly, derivative patterns above approximately 24.74 mm should be interpreted with caution because they are based on relatively sparse data and thus represent extrapolative behavior. Overall, these derivative curves provide visual evidence of transition tendency while also highlighting uncertainty in precise turning-point localization.

### 3.6 Exploratory turning-point estimation

**FIGURE 7:**
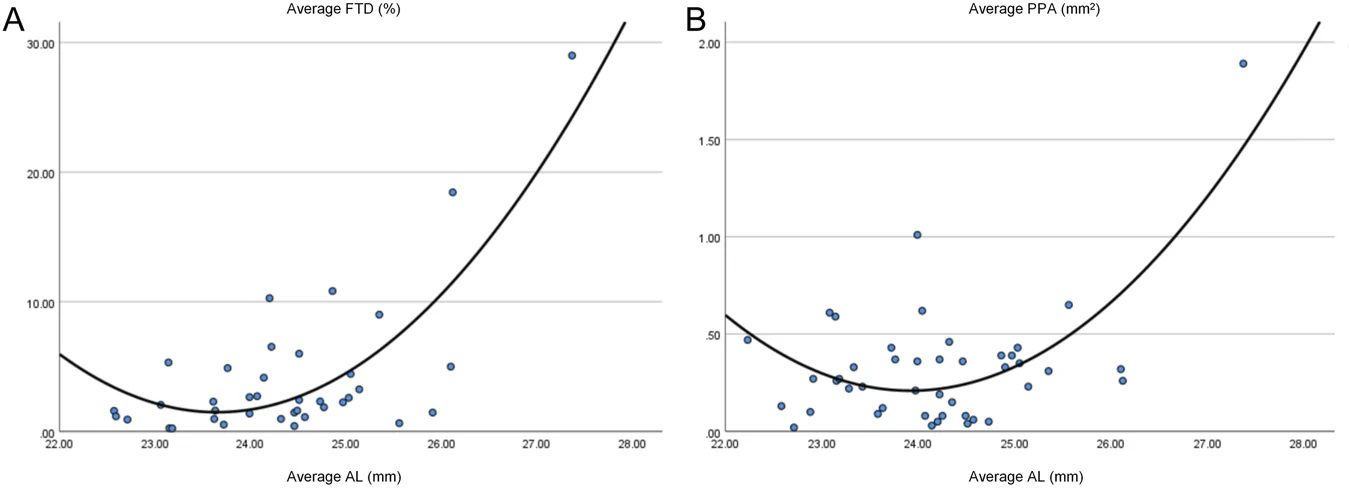
Nonlinear modeling of fundus structural parameters versus axial length. **(A)** FTD vs. AL; **(B)** PPA vs. AL. Quadratic regression (Y = *β*_*0*_ + *β*_*1*_ × AL + *β*_*2*_ × AL^2^) was applied to cross-sectional cohort data. Solid black curves represent fitted functions; gray shading indicates 95% confidence bands. FTD followed a statistically significant U-shaped trajectory (*β*_*2*_ = 0.182, *p* < 0.001), with vertex at AL = 23.66 mm (95% CI: 23.19–24.13 mm). PPA exhibited a J-shaped, accelerating relationship (*β*_*2*_ = 0.094, *p* < 0.001), with inflection point at AL = 23.99 mm (95% CI: 23.49–24.51 mm). These concordant inflection zones define a pediatric-specific biomechanical transition threshold at ∼24.0 mm.

While linear mixed-effects models confirmed a robust positive association between AL and PPA area, the AL–FTD relationship exhibited marked heterogeneity—suggesting stage-dependent, nonlinear dynamics rather than uniform linear progression. To formally test for nonlinearity, we fitted a quadratic regression model with AL and AL^2^ as predictors (Figure 7). The FTD–AL relationship was best described by a U-shaped quadratic function:

$$\text{FTD}=1.643\times {\text{AL}}^{2}-77.729\times \text{AL}+920.588$$

The estimated inflection point—the AL value at which the curvature changes sign—was 23.66 mm (95% CI: 23.19–24.13 mm). Below this threshold, FTD declined gradually with increasing AL (*β*_*1*_ = −77.729 + 2 × 1.643×AL < 0), consistent with physiological choroidal thickening or RPE–Bruch’s membrane compaction during early axial elongation. Above 23.66 mm, the second derivative becomes positive, and FTD rises steeply (*β*_*1*_ > 0), reflecting progressive choroidal thinning, vascular dropout, and loss of choriocapillaris integrity—hallmarks of incipient pathological remodeling. This inflection thus demarcates a biomechanical and microstructural transition from adaptive compensation to decompensated damage.

Similarly, the quadratic model for the PPA–AL relationship yielded:

$$\text{PPA}=0.105\times {\text{AL}}^{2}-5.037\times \text{AL}+60.445$$

The estimated inflection point was 23.99 mm (95% CI: 23.49–24.51 mm). Below this threshold, PPA area remained relatively stable and exhibited minimal change with AL elongation; above it, PPA expansion accelerated markedly—consistent with biomechanical failure of the peripapillary sclera, wherein tensile stress exceeds the tissue’s elastic limit and triggers irreversible remodeling. Critically, the inflection points for FTD (23.66 mm) and PPA (23.99 mm) were highly concordant (difference = 0.33 mm; 95% CI of difference: −0.12–0.78 mm), jointly identifying an anatomically and functionally coherent “transition zone” centered near 24.0 mm. This convergence strongly supports a shared pathophysiological threshold—beyond which cumulative axial elongation induces synchronous structural decompensation across multiple posterior pole compartments. Collectively, these findings demonstrate that PPA expansion undergoes a clear biomechanical tipping point, whereas FTD exhibits stage-specific, biphasic dynamics. Although cross-sectional modeling cannot substitute for longitudinal inference, it robustly complements the LMM results by uncovering the underlying nonlinear threshold effect that explains the heterogeneity observed in linear association estimates.

Table 3 summarizes the instantaneous rates of change (first derivatives) for FTD and PPA across clinically relevant AL intervals, along with their pathophysiological interpretations. At AL = 22.0 mm, FTD exhibited a steep negative slope (−5.44%/mm), consistent with physiological choroidal thickening—a compensatory response that reduces visibility of deep retinal vessels on fundus imaging. Concurrently, PPA showed minimal expansion (−0.39 mm^2^/mm), indicating structural stability in the peripapillary region. At the FTD inflection point (AL = 23.66 mm), the FTD derivative approached zero (−0.02%/mm), marking the nadir of the U-shaped trajectory and the onset of transition from adaptive compensation to early decompensation. At this same AL, PPA’s derivative was −0.08 mm^2^/mm—near its own inflection threshold—suggesting imminent biomechanical strain accumulation at the optic nerve head. At the PPA inflection point (AL = 23.99 mm), FTD’s derivative turned positive (+1.35%/mm), confirming the initiation of pathological choroidal thinning and microvascular dropout. Simultaneously, PPA’s derivative crossed zero (+0.01 mm^2^/mm), signifying the onset of accelerated scleral remodeling and irreversible peripapillary atrophy expansion. Beyond AL = 25.0 mm, both metrics demonstrated progressive acceleration: FTD increased at +4.39%/mm and PPA at +0.21 mm^2^/mm—indicating synergistic deterioration of chorioretinal and scleral integrity. At AL = 26.0 mm, these rates further escalated to +7.71%/mm and +0.42 mm^2^/mm, respectively, reflecting advanced posterior pole pathology characterized by rapid choroidal atrophy and expansive peripapillary tissue loss.

**TABLE 3:** Rates of change of FTD and PPA stratified by axial length (AL) and their clinical implications.

AL value (mm)	FTD change rate (%/mm)	Clinical interpretation of FTD	PPA change rate (mm^2^/mm)	Clinical interpretation of PPA
22.0	−5.44	Negative value, significant decrease. For every 1 mm increase in AL around 22 mm, FTD decreases by an average of 5.44%, indicating the eye remains in a physiological state in early myopia. To adapt to physiological AL elongation and regulate refractive status, compensatory physiological thickening of the choroid occurs, leading to reduced FTD.	−0.39	Negative value, slow change. PPA area atrophies or expands extremely slowly
23.66	≈0.00	Zero value, critical point. The first-order derivative at this point is zero, marking the transition of FTD from “decreasing” to “increasing”	−0.08	Still negative but close to zero; PPA atrophy is about to enter the acceleration phase
23.99	+1.35	Positive value, initial increase. After exceeding the FTD inflection point, the change rate turns positive. For every 1 mm increase in AL around 24 mm, FTD increases by an average of 1.35%, indicating the onset of pathological elevation	≈0.00	Zero value, critical point, marking the transition of PPA area from “slow change” to “accelerated atrophy”
25.0	+4.39	Positive value, rapid increase. Within the pathological myopia range, the growth rate of FTD with AL accelerates, indicating exacerbated pathological changes in the posterior pole (e.g., edema, proliferation)	+0.21	Positive value, accelerated atrophy. After exceeding the inflection point, PPA area expands by an average of 0.21 mm^2^ per 1 mm increase in AL, with significantly accelerated atrophy
26.0	+7.71	Positive value, sharp increase. At longer axial lengths, the growth rate of FTD rises sharply, reflecting severe pathological changes	+0.42	Positive value, high-speed atrophy. The atrophy rate doubles compared with that at AL of 25 mm, indicating lesions have entered the rapid progression stage

### 3.7 Robustness assessment of the 24.0-mm axial length threshold

**FIGURE 8:**
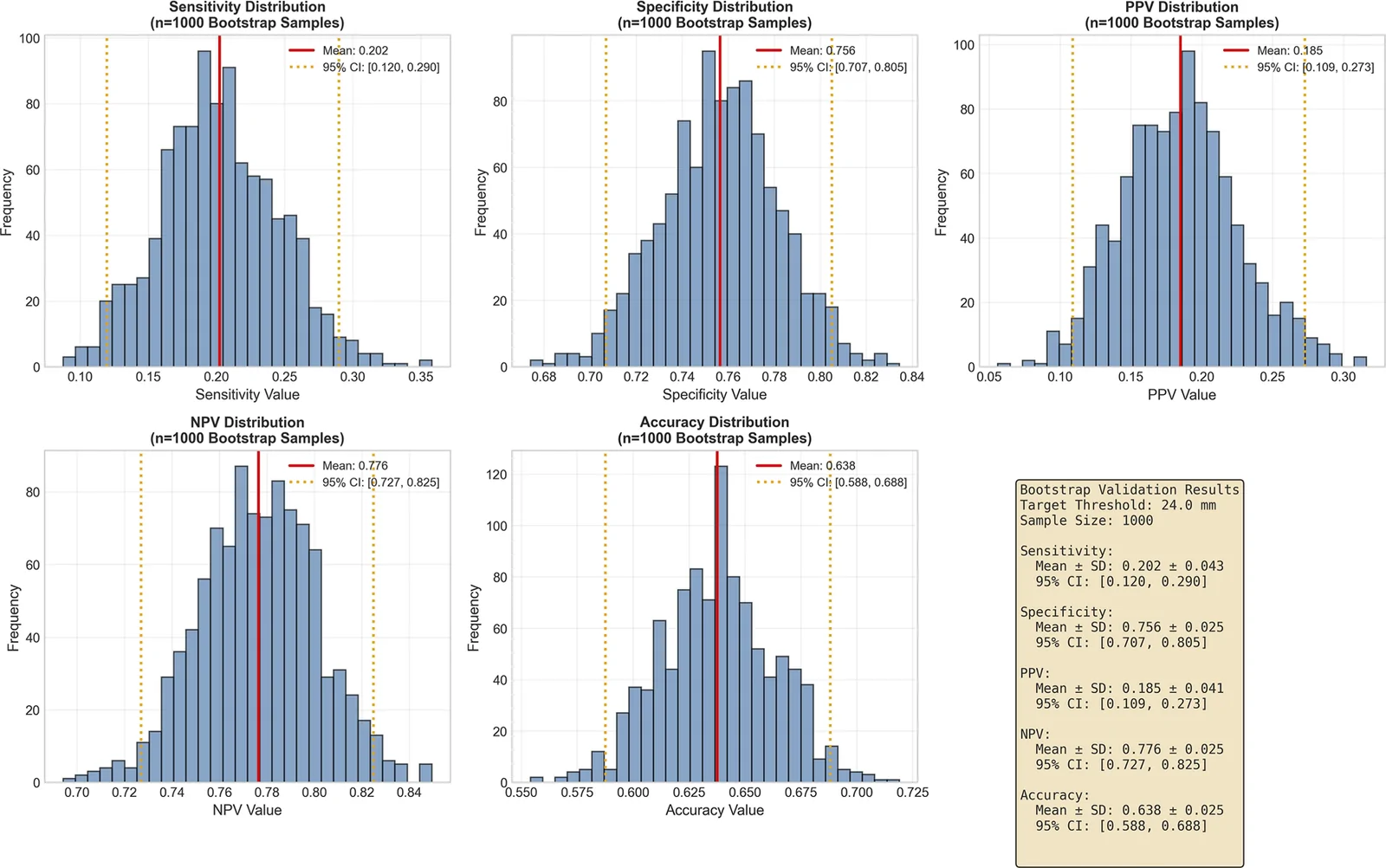
Bootstrap validation of the 24.0-mm axial length inflection zone. Distribution of estimated inflection points for fundus tessellation density (FTD) and peripapillary atrophy (PPA) across bootstrap resamples (n = 371 children). The FTD inflection point was centered at 23.66 mm (95% CI: 23.19–24.13 mm), and the PPA inflection point at 23.99 mm (95% CI: 23.49–24.51 mm). The substantial overlap of 95% confidence intervals confirms the statistical robustness and reproducibility of the convergent ∼24.0-mm biomechanical inflection zone for early posterior pole remodeling. x-axis: axial length (mm); y-axis: estimated inflection point density (arbitrary units). Solid vertical lines indicate median inflection points; shaded areas represent 95% bootstrap confidence intervals.

To evaluate the stability of the identified AL threshold (∼24.0 mm) across sampling variability, internal validation was performed using bootstrap resampling (1,000 iterations with replacement). For each bootstrap sample, the quadratic regression models for FTD and PPA were refitted independently, and the corresponding inflection points—defined as the AL values at which the second derivative equals zero—were computed. Distributional properties (mean, 95% confidence interval, and coefficient of variation) of the bootstrapped inflection estimates are summarized in Figure 8. The bootstrap-derived mean inflection point for FTD was 23.66 mm (95% CI: 23.19–24.13 mm; CV = 10.2%), and for PPA it was 23.99 mm (95% CI: 23.49–24.51 mm; CV = 11.5%). Both coefficients of variation fall within the empirically accepted range for stable nonlinear parameter estimation (<15%). Critically, the 95% CIs of the two inflection points overlap substantially (23.19–24.13 mm vs. 23.49–24.51 mm), confirming their statistical concordance. These results demonstrate that the ∼24.0-mm threshold is highly reproducible across resampled datasets, exhibits low sensitivity to outlier influence or finite-sample fluctuations, and thus constitutes a statistically robust and clinically actionable cut-off for differentiating physiological ocular growth from early pathological fundus remodeling in adolescent myopia.

### 3.8 Sex-stratified nonlinear analysis

In sex-interaction models, the joint LRT for AL-related interaction terms was non-significant for FTD (χ^2^ = 1.782, df = 2, *P* = 0.410) but significant for PPA (χ^2^ = 10.128, df = 2, *P* = 0.0063) (Supplementary Table S3).

In sex-stratified quadratic models, the raw turning points were 21.71 mm (female) and 22.38 mm (male) for FTD, and 22.54 mm (female) and 23.13 mm (male) for PPA.

Bootstrap medians (95% CI) were 22.03 (11.17–29.10) and 22.48 (17.19–23.16) mm for FTD (female and male), and 21.99 (20.76–22.49) and 22.73 (21.39–23.25) mm for PPA.

The bootstrap median between-sex difference (male–female) was 0.42 mm (95% CI: −14.27–16.92) for FTD and 0.75 mm (95% CI: −0.57–2.02) for PPA, indicating no statistically robust difference in turning point location (Supplementary Table S3; Supplementary Figure S3).

Sensitivity analyses showed limited contribution of sex terms for FTD (AIC: 4680.62, 4680.14, 4682.36 for m0, m1, m2), whereas PPA model fit improved when sex interactions were included (AIC: −398.61, −400.07, −406.20).

### 3.9 Percentile contextualization of AL = 24.0 mm

In age- and sex-specific Chinese normative data, the percentile corresponding to AL = 24.0 mm varied across strata. In girls, 24.0 mm corresponded approximately to the 80.second, 70.4th, and 59.8th percentiles at ages 9, 10, and 11 years, respectively; in boys, corresponding values were 60.0th, 50.4th, and 41.4th percentiles. In the longitudinal follow-up visits of our cohort, the proportions with AL ≥ 24.0 mm were 9.0% (13/145), 24.5% (62/253), and 26.7% (27/101) for girls aged 9, 10, and 11 years, and 40.9% (79/193), 46.5% (140/301), and 54.5% (61/112) for boys of the same ages (Supplementary Table S1). These results indicate substantial age- and sex-specific heterogeneity in the interpretation of a fixed AL value (Supplementary Figure S2).

## 4 Discussion

This study employed a 2-year prospective longitudinal design with annual standardized ophthalmic assessments to characterize the nonlinear evolution of fundus microstructures during adolescent myopia progression. Its principal contributions are twofold (Holden et al., 2016): Using AI-powered quantitative imaging, we established FTD and PPA as robust, mechanistically grounded biomarkers—demonstrating that FTD reflects stage-specific choroidal adaptation (U-shaped trajectory), while PPA quantifies cumulative peripapillary scleral strain (accelerating expansion beyond transition zone); and (Vagge et al., 2026) Within a rigorously phenotyped longitudinal cohort, we identified a convergent biomechanical inflection zone centered at 24.0 mm (95% CI: 23.66–24.51 mm), which marks the transition from compensatory ocular growth to incipient posterior pole pathology—a finding validated by bootstrap resampling and consistent across independent structural endpoints.

Prior studies have predominantly characterized tessellated fundus using qualitative grading systems or semiquantitative scales—approaches inherently limited in resolving fine-grained, time-dependent structural dynamics during myopia progression (Huang et al., 2023a; Guo et al., 2024). Huang et al. demonstrated that AI-quantified FTD is significantly inversely correlated with subfoveal choroidal thickness, supporting its biological relevance to choroidal integrity. Building on this, our longitudinal analysis reveals a substantially amplified FTD differential: participants with long axial length (AL > 26.0 mm) exhibited median FTD values over fivefold higher than those with short AL (AL < 24.0 mm) (*p* < 0.001), and FTD increased progressively across follow-up visits—consistent with progressive choriocapillaris attenuation and RPE–Bruch’s membrane disruption. Furthermore, cohort studies of early-onset high myopia corroborate FTD’s strong association with AL elongation (Guo et al., 2025; Moon and Lim, 2021). Crucially, our temporally resolved data establish the directional sequence: AL elongation consistently precedes or co-occurs with rising FTD, reinforcing FTD’s utility as a sensitive, noninvasive biomarker of axial strain–induced choroidal microstructural deterioration. Most notably, this study refutes the prevailing assumption of monotonic FTD increase with AL. Instead, we provide robust empirical evidence—validated by quadratic modeling and bootstrap stability assessment—that FTD follows a U-shaped, biphasic trajectory. Below the inflection point (AL < 23.66 mm), FTD declines with AL elongation, reflecting physiological choroidal thickening; above it, FTD rises steeply, marking the transition to pathological atrophy (Guo et al., 2025; Moon and Lim, 2021). The underlying mechanism reflects distinct phases of choroidal response to axial elongation. During emmetropization in early childhood, the choroid undergoes physiological thickening—a compensatory adaptation that reduces visibility of deep retinal vessels on fundus imaging and supports coordinated, homeostatic ocular growth. In contrast, once AL exceeds 23.66 mm—the empirically derived inflection transition zone—FTD rises steeply, indicating a shift from adaptive compensation to biomechanical decompensation. At this stage, excessive axial elongation surpasses the elastic reserve of the choroid and peripapillary sclera, resulting in choriocapillaris compression, stromal atrophy, and progressive exposure of deeper vascular layers. These findings establish FTD, quantified from widely available color fundus photographs, as a scalable, noninvasive surrogate marker of choroidal microstructural integrity. Its clinical utility is particularly salient in resource-constrained settings, where optical coherence tomography (OCT) remains inaccessible for population-level myopia screening.

Regarding the association between PPA and myopia progression, prior evidence remains heterogeneous. Moon and Lim (2021) reported that smaller baseline *β*-zone PPA area was associated with faster subsequent myopia progression—a finding interpreted as reflecting reduced biomechanical reserve. In contrast, a cohort study of children aged 8–11 years demonstrated progressive *β*-PPA expansion with increasing AL, suggesting PPA may instead serve as a marker of cumulative scleral deformation (Zhang et al., 2022). Similarly, longitudinal data from another investigation linked larger baseline PPA area to greater subsequent thinning of macular choroidal thickness, implicating shared pathophysiological pathways across posterior pole compartments (Li et al., 2024). Our longitudinal analysis clarifies this discrepancy: unlike FTD—which follows a U-shaped, stage-dependent trajectory—PPA exhibits monotonic, cumulative expansion across the entire AL range. Critically, the annual AL growth rate showed a statistically significant positive correlation with annual PPA expansion (r = 0.324, *p* < 0.001), and AL remained a highly significant predictor in multivariable linear mixed-effects models (*β* = 0.127 mm^2^/mm, *p* < 0.001). These consistent, dose-dependent associations robustly support PPA as a quantitative biomechanical indicator of peripapillary scleral strain—directly validating the mechanical stretching hypothesis (Dai et al., 2024; Yin and Ge, 2025). Notably, a recent large-scale prospective study in Chinese children identified 0.20 mm/year as a clinically meaningful cut-off for axial elongation rate, distinguishing non-myopic children exhibiting an “obvious myopic shift” from those maintaining stable refraction (Liu et al., 2025). In our cohort, the stable progression group’s mean AL growth rate (0.147 mm/year) fell below this transition zone, whereas the moderate (0.44 mm/year) and rapid (0.83 mm/year) groups significantly exceeded it—concurrently demonstrating accelerated PPA expansion. This tight coupling between AL velocity and PPA dynamics reinforces axial elongation as the primary biomechanical driver of peripapillary structural damage.

Mechanistic basis for the divergent functional forms. The contrasting shapes are consistent with the different tissue processes that the two biomarkers report. FTD is an optical visibility metric: it reflects the extent to which large choroidal vessels are discernible through the overlying RPE, and is therefore governed jointly by choroidal thickness, choroidal vascular calibre and RPE pigment density, all of which are bidirectionally modifiable (Xue et al., 2024; Zhou et al., 2018; Venkatesh et al., 2026). In non-myopic and slowly elongating childhood eyes the choroid physiologically thickens, reducing vessel visibility and generating the descending limb (AL < 23.66 mm); once elongation outpaces the choroid’s capacity to accommodate it, choriocapillaris compression and stromal thinning supervene, visibility rises steeply, and the ascending limb emerges (Jin et al., 2019; Read et al., 2015; Uzun and Pehlivan, 2016). A U-shaped function is thus the expected signature of a reversible biomarker governed by two competing processes whose net balance changes sign. PPA, by contrast, is a cumulative tissue-displacement metric, quantifying the region from which the RPE–Bruch’s membrane complex has retreated, or which it never reached, following the temporal shift and later enlargement of the Bruch’s membrane opening during axial elongation (Jonas et al., 2017; Wang et al., 2021). Because this displacement is not reversed over the timescale of childhood, absolute PPA area can only remain stable or increase, which precludes a U shape (Lee et al., 2018; Kim et al., 2018). Below the transition point, peripapillary strain remains within the quasi-linear range of the scleral collagen matrix and the crescent changes little; beyond it, non-linear scleral creep and collagen reorganisation predominate and area accrues at an accelerating rate, producing the J shape (McBrien et al., 2009; Boote et al., 2020). The 0.33-mm offset between transition points corresponds to approximately 9 months at the mean elongation rate of the moderate-progression group (0.44 mm/year), suggesting that choroidal decompensation marginally precedes accelerated peripapillary remodelling rather than the two arising independently—consistent with cross-sectional reports that greater FTD and larger PPA co-occur (Huang et al., 2023b).

During childhood, the sclera remains in an active developmental remodeling phase, characterized by high extracellular-matrix (ECM) plasticity, dynamic collagen turnover, and lower effective biomechanical stiffness than in mature adult eyes. Under sustained axial elongation, this developmental tissue state is more prone to creep-related deformation and earlier posterior-pole biomechanical decompensation (Rada et al., 2006; McBrien et al., 2009; Boote et al., 2020). This provides a biologically plausible explanation for why the structural transition identified in our cohort occurs near AL ≈ 24 mm, below the adult high-myopia benchmark (AL > 26 mm) (Rada et al., 2006; McBrien et al., 2009). Importantly, these values are not directly equivalent: the adult benchmark is a late risk classifier, whereas our finding reflects a pediatric growth-phase transition zone in structural remodeling dynamics.

Conventionally, an AL exceeding 26.0 mm in adulthood is widely regarded as a clinical transition zone for high myopia and associated pathological risk (Haarman et al., 2020; Tideman et al., 2016). However, our longitudinal data from children aged 8–11 years—during peak ocular growth plasticity—demonstrate that structural decompensation at the posterior pole initiates substantially earlier than this adult-derived benchmark. Quadratic modeling of FTD and PPA trajectories converged on a pediatric-specific biomechanical inflection zone centered at 24.0 mm (FTD inflection: 23.66 mm; PPA inflection: 23.99 mm; 95% CIs overlapping), marking the transition from adaptive growth to incipient pathological remodeling. This 24.0-mm transition zone holds direct clinical utility for early risk stratification. When contextualized within age- and sex-specific AL percentile curves for Chinese children, an AL of 24.0 mm frequently corresponds to the 75th–95th percentile—indicating markedly accelerated growth relative to peers and conferring elevated risk for progressive fundus changes (He X. et al., 2023). Our supplementary percentile contextualization shows that a fixed AL value of 24.0 mm does not represent the same developmental position across pediatric subgroups. In girls aged 9–11 years, 24.0 mm generally lies above the median and up to the upper-percentile range, whereas in boys of similar ages it is closer to the median or below the age-specific median at older ages. Therefore, 24.0 mm should be interpreted as a candidate transition zone requiring age- and sex-specific clinical context, rather than a universal cutoff. Critically, the close concordance between the two inflection points (difference = 0.33 mm; 95% CI: −0.12–0.78 mm) supports synchronous onset of choroidal microvascular disruption (reflected by rising FTD) and peripapillary scleral failure (reflected by accelerating PPA expansion). Thus, 24.0 mm represents a biomechanical tipping point in the developing eye—where the combination of inherent tissue plasticity and excessive elongation velocity generates sufficient tensile stress to compromise choriocapillaris perfusion and induce marginal optic disc tissue separation. This interpretation aligns with emerging clinical evidence: even in the absence of conventionally defined high myopia (AL < 26.0 mm), progressive axial elongation independently predicts increased risk of retinopathy and functional visual impairment. A recent multicenter systematic review confirmed that cumulative risk of vision-threatening complications rises continuously with increasing AL—without evidence of a lower transition zone effect (Shah et al., 2024; Shu et al., 2024). Furthermore, operationalizing the 24.0-mm inflection transition zone carries direct implications for clinical decision-making and intervention timing. Prior evidence indicates that while increased outdoor time robustly reduces myopia incidence in premyopic children, its efficacy diminishes markedly once axial elongation enters the accelerated phase—exhibiting a nonlinear attenuation of protective effect. Consequently, when a child’s axial length approaches the 24.0-mm inflection zone (e.g., AL ≥ 23.5 mm with documented annual growth >0.20 mm/year), clinicians should transition from passive behavioral counseling to proactive, mechanism-targeted interventions. These may include low-concentration atropine (0.01%–0.05%) or optical defocus–modifying spectacles—therapies with demonstrated efficacy in slowing axial progression and mitigating posterior pole structural deterioration. Early intervention at this biomechanically defined inflection point aims not merely to reduce refractive error progression, but to preserve chorioretinal integrity and prevent irreversible microstructural damage (He et al., 2015; Shih et al., 2016; Chen et al., 2026).

The clinical implication is that interventions that slow axial elongation may also reduce cumulative posterior-pole structural load. Low-dose atropine and defocus-based spectacle strategies have shown efficacy in reducing myopia progression/AL growth in children (Lam et al., 2020; Ya et al., 2020); therefore, they are mechanistically expected to delay or attenuate transition toward maladaptive choroid–sclera remodeling (Chun et al., 2023). However, this structural-protection interpretation should be considered translational and hypothesis-guided: current intervention trials are primarily powered for refractive/AL outcomes, and direct validation using prespecified structural endpoints (e.g., AI-quantified FTD and PPA trajectories) remains limited (Brennan et al., 2021). We therefore propose future prospective trials to include FTD/PPA as scalable structural outcome measures.

**FIGURE 9:**
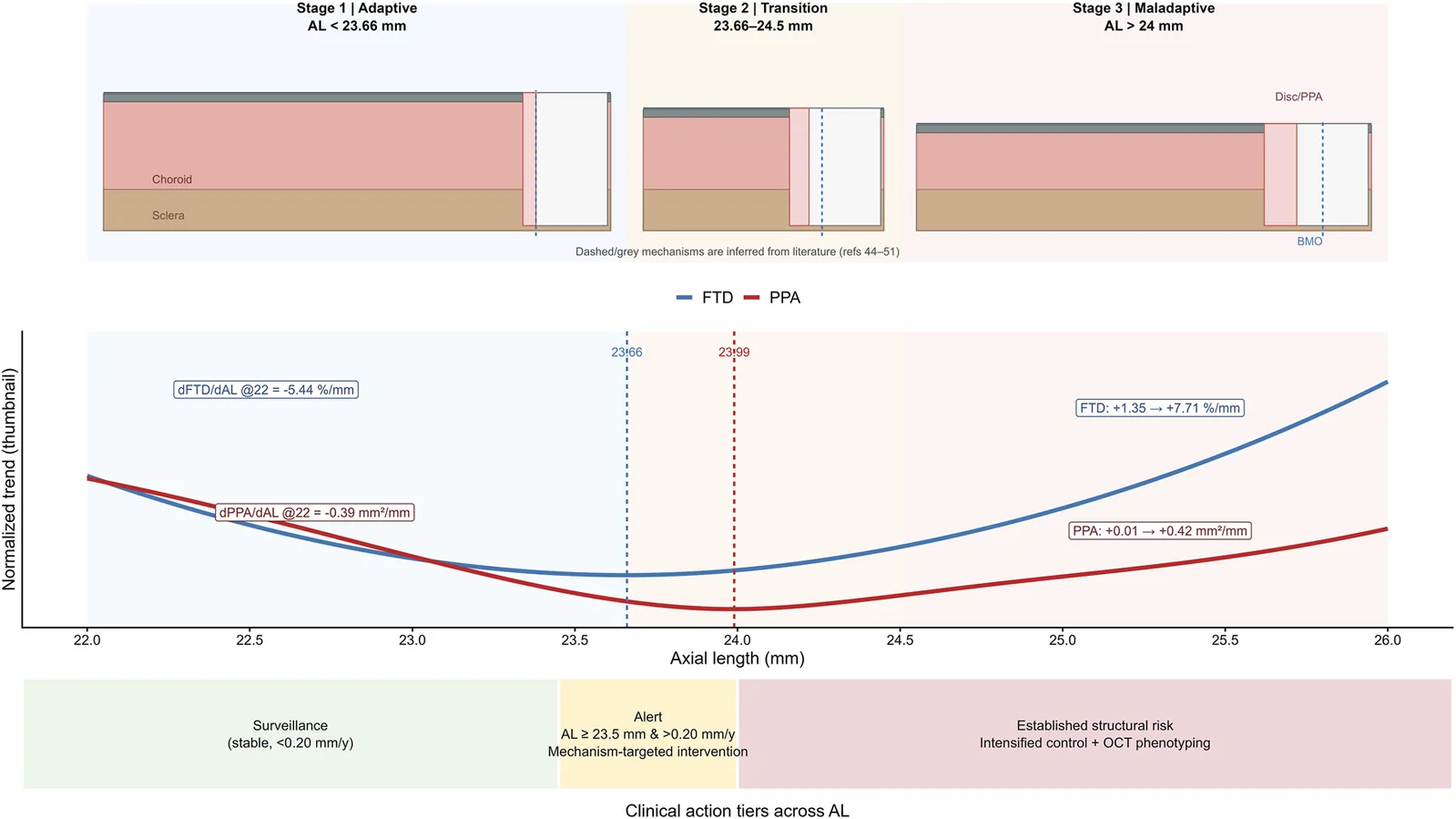
Proposed cascade of posterior-pole remodelling across the ∼24-mm axial transition zone in school-aged children. Solid elements (AL, FTD, PPA and their first derivatives, with values from Table 3; Figures 7, 8; n = 371 children) were measured in the present cohort; dashed/grey elements (scleral extracellular-matrix creep and collagen reorganization, choriocapillaris compression, RPE attenuation, Bruch’s-membrane-opening displacement) denote mechanisms inferred from published histological, biomechanical and OCT evidence and are hypothetical in this cohort. Stage 1 (AL < 23.66 mm): compensatory choroidal thickening reduces large-vessel visibility (dFTD/dAL = −5.44%/mm at AL 22.0 mm) while peripapillary strain remains within the quasi-linear elastic range (dPPA/dAL = −0.39 mm^2^/mm). Stage 2 (23.66–23.99 mm, ≈9 months at the moderate-group elongation rate of 0.443 mm/y): both derivatives cross zero, marking exhaustion of adaptive reserve. Stage 3 (AL > 24 mm): FTD and PPA accelerate in parallel (+1.35 to +7.71%/mm and +0.01 to +0.42 mm^2^/mm), consistent with combined choroidal microvascular decompensation and non-linear peripapillary scleral remodelling. The lower bar indicates the corresponding proposed tiers of clinical management.

This study underscores the clinical imperative of identifying children at elevated risk for rapid myopia progression at the earliest feasible stage. Cluster analysis stratified participants into progression phenotypes, with the “rapidly progressive” group—defined by annual AL growth exceeding 0.80 mm/year—demonstrating significantly higher prevalence of early fundus structural pathology (*p* < 0.001). These findings provide an evidence-based framework for risk-stratified management: children exhibiting slow, linear AL growth and stable fundus microstructure may be managed with routine surveillance; in contrast, those with accelerated AL elongation concurrent with emerging FTD elevation or PPA expansion warrant prompt, intensified intervention to avert transition to pathological myopia. Critically, this work integrates AI-quantified fundus biomarkers—FTD and PPA—into the longitudinal monitoring paradigm. Whereas conventional clinical assessment relies exclusively on refractive error and AL measurements, our approach objectively captures subclinical microstructural alterations using deep learning–driven image analysis. AI quantification delivers three key advantages over manual grading: (i) objectivity—eliminating subjective interpretation bias; (ii) intra- and inter-rater consistency—essential for multicenter reproducibility; and (iii) quantitative precision—enabling detection of subtle, progressive changes (e.g., diffuse tessellation gradation or irregular PPA margin delineation) beyond the resolution of unaided human assessment. This standardization directly mitigates inter-observer variability—a major limitation in large-scale epidemiological and screening studies (Figure 9).

This study has several methodological limitations. First, the cohort was recruited exclusively from schools in Guangzhou, a metropolitan region with distinct environmental exposures—including high academic pressure, limited outdoor time, and predominantly Han Chinese ethnicity—potentially limiting the generalizability of findings to other demographic or geographic populations. Furthermore, gender-stratified analysis is exploratory in nature, and the current sample size yields limited statistical power to detect “minor curvature differences”. Accordingly, the 24.0-mm turning point identified in this cohort should be interpreted as internally valid but externally unverified, and should not be directly extrapolated to other ethnic, geographic, or age groups until independently replicated. External validation is currently being conducted in the multicenter China Myopia Progression Study (CMPS). Second, segmentation was performed with a commercial platform whose development, annotation protocol and test-set performance have been published (He et al., 2024; Long et al., 2022; He H et al., 2023); we applied the pretrained model unchanged and did not recompute Dice/IoU on a newly annotated subset of the present cohort, assessing instead agreement between AI output and expert consensus in a random 10% sample (ICC ≥0.92). Reported test-set metrics for tessellation detection were expressed as per-pixel accuracy, sensitivity and specificity rather than Dice/IoU, and direct comparison with region-overlap metrics should therefore be made with caution. Third, the 2-year follow-up duration captures only short-to medium-term structural dynamics; longer-term observation is required to determine whether FTD elevation and PPA expansion predict clinically significant endpoints of pathological myopia, such as macular atrophy, choroidal neovascularization (CNV), or permanent visual impairment. Fourth, while the U-shaped trajectory of FTD is robustly modeled, the biological basis of its descending limb (AL < 23.66 mm) remains incompletely characterized; integration with longitudinal OCT-derived choroidal thickness and vascular density metrics will be essential to distinguish physiological thickening from early microvascular remodeling. Fifth, PPA was quantified as the total peripapillary atrophic crescent area rather than as separate α-, β-, and γ-zones. Reliable differentiation between β- and γ-zones requires OCT-based visualization of the presence/termination of Bruch’s membrane (BM) and the termination of the retinal pigment epithelium (RPE); therefore, zone-specific attribution cannot be reliably performed on color fundus photographs alone. Future CMPS analyses will incorporate OCT to enable zonal PPA phenotyping. In response, we have initiated a national multicenter prospective cohort—the CMPS—enrolling over 15,000 children across 11 provinces and municipalities. This expanded study integrates multimodal data: genome-wide single nucleotide polymorphism (SNP) profiling, real-time environmental exposure monitoring (e.g., light intensity, near-work duration), and advanced ocular imaging (spectral-domain OCT, OCT angiography, and ultra-widefield fundus photography). Its primary objectives are threefold: (i) to externally validate the 24.0-mm biomechanical inflection zone in ethnically and environmentally diverse populations; (ii) to refine a dynamic, personalized risk prediction model for progression phenotypes by incorporating genetic susceptibility, environmental load, and AI-quantified structural biomarkers; and (iii) to translate this evidence into clinical practice through development of an AI-assisted decision support tool for tiered intervention—aiming to reduce the incidence of irreversible vision loss attributable to pathological myopia (He et al., 2015).

## 5 Conclusion

Based on a 2-year prospective longitudinal cohort with annual standardized ophthalmic assessments, this study delineates the nonlinear, stage-specific evolution of FTD and PPA in relation to AL elongation during adolescent myopia progression—using AI-powered quantitative analysis of color fundus photographs. Within a rigorously phenotyped longitudinal cohort, we identified a convergent biomechanical inflection zone centered at 24.0 mm (95% CI: 23.66–24.51 mm), which represents the critical transition point from adaptive ocular growth to incipient posterior pole pathology. Below this zone (AL < 23.66 mm), FTD declines progressively—consistent with physiological choroidal thickening that attenuates deep vessel visibility. Above it (AL > 23.99 mm), FTD rises steeply while PPA exhibits accelerating, J-shaped expansion—collectively signaling biomechanical failure of the posterior sclera–choroid complex and irreversible microstructural decompensation. Cluster analysis stratified progression phenotypes using annual AL growth rate, identifying a “rapid progression” subgroup defined by ≥ 0.80 mm/year. This phenotype is characterized by three interdependent features: elevated AL, accelerated elongation velocity, and synchronous deterioration of FTD and PPA—constituting a high-risk structural signature warranting early, mechanism-targeted intervention. Critically, these findings supersede the conventional adult-derived high-myopia transition zone (AL > 26.0 mm) for risk stratification. Instead, they establish pediatric-specific, imaging-anchored quantitative criteria—validated by bootstrap stability and longitudinal concordance—that enable earlier, more precise identification of children at risk for pathological myopia. This advances clinical practice from reactive refractive management toward proactive, biomarker-guided prevention and personalized intervention in school-aged populations.

## Data Availability

The raw data supporting the conclusions of this article will be made available by the authors, without undue reservation.
