# Supplementary material for: Defining the critical axial length threshold for transition from physiological growth to pathological fundus structural remodeling in adolescent myopia
Source: Front Cell Dev Biol. 2026 Sep 4;14:1938535. doi: 10.3389/fcell.2026.1938535 (PMC13585632; doi:10.3389/fcell.2026.1938535)
Supplement: Supplementary file 1 [file DataSheet1.zip › Supplementary Material/Supplementary Figure/Supplementary Figure legend.docx]

Supplementary Material

Supplementary Figure legend：

Supplementary Figure S1. Representative color fundus images showing AI segmentation overlays for FTD, PPA, and the original image.

Supplementary Figure S2. Age-specific pediatric axial length percentile context for AL = 24.0 mm. Normative curves (P50, P75, P90, P95) were redrawn from published Chinese numerical reference tables. The dashed horizontal line indicates AL = 24.0 mm. Points represent visit-level observations in the study cohort.

Supplementary Figure S3 Sex-stratified analyses of quadratic associations between axial length and FTD/PPA. (A) Fitted quadratic curves with raw turning points (vertical dashed lines) for females (red) and males (blue). (B) Bootstrap distributions of turning points with 95% CIs and between-sex differences. The sex-interaction LRT was significant for PPA (*P*=0.0063) but not for FTD (P=0.410). Abbreviations: AL, axial length; CI, confidence interval; FTD, fovea-to-disc distance; LRT, likelihood ratio test; PPA, peripapillary atrophy.
